# Dental Supplies

**Published:** 1862-04

**Authors:** 


### DENTAL SUPPLIES.

OUR friend Jno. Nash, present proprietor of the Toland dental depot, is manifesting the same perseverance in providing for the requirements of the dental profession that characterized the former proprietor.

All dental materials can be obtained there, and upon terms of which none can complain. The immediate management of the business is in charge of Mr. E. T. Lovering, whose energy, ability and gentlemanly bearing are such as to win the high regard and confidence of all those with whom he does business. He has just returned from the East with a full supply of new stock.

